# Characterization of Newly Synthesized Nanobiomaterials for the Treatment of White Spot Lesions

**DOI:** 10.3390/biom16010112

**Published:** 2026-01-08

**Authors:** Andra Clichici, Diana Dudea, Cristina Gasparik, Camelia Alexandra Coadă, Ioana Bâldea, Stanca Cuc, Mărioara Moldovan

**Affiliations:** 1 Department of Prosthetic Dentistry and Dental Materials, Iuliu Hațieganu University of Medicine and Pharmacy, 400006 Cluj-Napoca, Romania; andra.clichici@umfcluj.ro (A.C.);; 2 Department of Physiology, Iuliu Hațieganu University of Medicine and Pharmacy, 400006 Cluj-Napoca, Romania; 3 Department Polymeric Composites, Raluca Ripan Chemistry Research Institute, Babes Bolyai University, 400294 Cluj-Napoca, Romania; stancabobo@yahoo.com (S.C.);

**Keywords:** infiltration resins, white spot lesions, demineralization lesions, cytotoxicity

## Abstract

Background: White spot lesions (WSLs) are characterized by enamel demineralization. Minimally invasive treatments using infiltrating resins, such as the commercially available Icon^®^, are recommended. The need for such treatments justifies ongoing research into developing materials that can address existing limitations regarding strength, durability, and biocompatibility. Objectives: This study aimed to synthesize and characterize four novel nanobiomaterials by evaluating their physicochemical properties and biocompatibility compared to the commercial material Icon^®^. Materials and methods: The recipes for the experimental nanobiomaterials NB3, NB6, NB3F, and NB6F contain varying proportions of TEGDMA, UDMA, HEMA, Bis-GMA, and HAF-BaF2 glass. Mechanical and physicochemical characteristics were evaluated, such as flexural strength, measured using the three-point test; water absorption and solubility; fluoride release; polymerization conversion; and residual monomers, assessed using High-Performance Liquid Chromatography (HPLC). In vitro cell viability was assessed via colorimetry using human dysplastic oral keratinocytes (DOKs). Results: NB6 and NB6F demonstrated the greatest polymerization potential. NB3 exhibited the lowest water absorption and solubility due to its hydrophobic nature. Additionally, the inclusion of UDMA enhanced the strength and elasticity of NB3 when compared to NB6. Among the samples with fluoride additives (NB3F and NB6F), the highest fluoride release on day 7 occurred with the material lacking UDMA. In contrast, the NB3F sample containing UDMA released the least amount of fluoride on the same day. In quantitative terms, NB3 and NB6F exhibited the lowest levels of residual monomers, whereas NB6 showed the highest levels. Both NB3 and NB6 were significantly better tolerated by the cells, showing higher cell viability compared to the commercial material Icon^®^. Conclusions: The materials’ mechanical and physicochemical properties varied with component proportions, enabling identification of a suitable formulation for targeted clinical applications. Biocompatibility tests showed that the experimental NB3 and NB6 were better tolerated than Icon^®^. Furthermore, the incorporation of filler particles improved the mechanical strength of the experimental nanobiomaterials.

## 1. Introduction

White spot lesions (WSLs) arise from an imbalance between demineralization and remineralization processes in the oral cavity, influenced by factors such as bacterial plaque accumulation due to poor oral hygiene or orthodontic treatment [1,2]. If left untreated, this type of early enamel demineralization can progress to cavitated lesions, presenting both functional and aesthetic concerns for patients, as the lesion and its progression can affect the appearance of their teeth [3].

The treatment of these incipient lesions involves infiltration resins or remineralizing agents. Both methods are minimally invasive and aim to preserve hard dental tissue. Infiltration resins, known for their low viscosity and density, penetrate the porous, demineralized enamel to seal the affected area. This process inhibits the proliferation of cariogenic bacteria and their acid by-products, thereby reducing the risk of carious progression and secondary caries [4].

A currently available material for addressing the effects of white spot lesions is Icon^®^ (DMG, Hamburg, Germany), which offers several advantages, including enhancement of microhardness in areas of enamel demineralization, the ability to complete treatment in a single, painless session, and the possibility to halt caries progression. Furthermore, it postpones the need for restorative treatment, reduces the risk of recurrent caries, and minimizes the risk of pulp inflammation and postoperative sensitivity. The likelihood of periodontitis and gingivitis is also decreased, while aesthetic outcomes improve by concealing demineralized enamel [5].

The composition and component ratios in infiltration materials can influence their mechanical, physicochemical, and biocompatibility properties. The main component of Icon^®^ resin, triethylene glycol dimethacrylate (TEGDMA), is a low-viscosity monomer with a high water sorption rate, which may lead to discoloration [5,6]. Studies have also shown that the addition of TEGDMA results in limited mechanical strength, with significantly lower flexural strength and modulus of elasticity than other dental materials, potentially compromising its durability in the oral cavity [7,8]. Enhanced mechanical properties of infiltration resins reduce the risk of wear caused by factors such as tooth brushing. Additionally, superior mechanical properties can reinforce demineralized lesions, potentially improving their resistance to further degradation [9].

The biocompatibility of Icon^®^ resin raises concerns due to residual monomers, which could trigger biological reactions and potentially compromise its long-term safety and efficacy in clinical use [5]. A recent study [7] synthesized and characterized novel experimental resin infiltrants (based on TEGDMA, HEMA, UDMA, Bis-GMA, and BaF2-based glass), evaluating their degree of conversion (DC), water absorption, microstructure, fluoride release, flexural strength, Young’s modulus, and residual monomer content. These materials exhibited promising physicochemical and mechanical properties.

When used on demineralized enamel, fluorohydroxyapatite and barium fluoride (BaF_2_) glass effectively inhibit caries progression and support remineralization. Fluorohydroxyapatite nanoparticles create a bioactive layer that closely resembles natural enamel [10]. Their use in dental materials, especially combined with BaF_2_ glass fillers, has been extensively studied, showing high remineralizing capacity, good coverage of WSLs, and effective infiltration. Besides their bioactivity, adding HAF–BaF_2_ glass fillers improves the mechanical properties of resin-based materials, increasing flexural strength, hardness, and wear resistance, which enhances durability [11,12]. When exposed to oral fluids, this mixture releases ions that promote hydroxyapatite crystal formation on the surface [13,14]. Research also indicates its effectiveness in treating erosion lesions, preventing demineralization, and aiding the remineralization of carious areas. Additionally, BaF_2_ glass offers broad-spectrum antimicrobial effects and improved radiopacity [7,15].

This study aimed to evaluate four experimental infiltration resins synthesized at the Raluca Ripan Institute of Chemistry, compared with the commercial material Icon^®^ (DMG, Hamburg, Germany).

In addition to assessing flexural strength, water absorption, fluoride release, polymerization conversion, and residual monomers (HPLC), this research incorporates biocompatibility testing of human dysplastic oral keratinocytes (DOK cells), providing a comprehensive analysis of both functional and biological properties.

## 2. Materials and Methods

For this study, four new nanobiomaterials with potential applications for treating WSLs were synthesized and characterized at the Raluca Ripan Institute of Chemistry Research in Cluj-Napoca. The radiopaque glass (BaF_2_), composed of SiO_2_, B_2_O_3_, Al_2_O_3_, and BaF_2_, was synthesized using a conventional melting technique. Fluorohydroxyapatite was synthesized via a precipitation method using calcium fluoride (CaF_2_) and phosphoric acid (H_3_PO_4_) as precursor materials. The fluorohydroxyapatite (HAF) and glass powders were subsequently mixed and co-sieved. Before use, the radiopaque glass powder was silanized with 3-methacryloxypropyltrimethoxysilane (silane A-174). The materials were labeled as NB3, NB3F, NB6, and NB6F (Table 1).

### 2.1. Flexural Strength

Five parallelepiped-shaped samples (24 × 2 × 2 mm) were fabricated for each material using a Teflon mold. The samples were light-cured for 20 s with an LED lamp (Guilin Woodpecker Medical Instruments Co., Guilin, China) at five different points. After curing, the samples were stored in distilled water at 37 °C for 72 h. Flexural strength was evaluated using a three-point bending test conducted according to ISO 4049/2000 international standard [16]. Testing was performed with a mechanical testing device (Ametek, Lloyd Instruments, Berwyn, PA, USA). The test parameters included a migration speed of 0.5 mm/min and a load of 50 N. The modulus of elasticity (Young’s Modulus) was determined using the NEXYGEN 4.18 software integrated with the universal testing machine, which continuously recorded the material’s response to the applied force throughout the testing process.

### 2.2. Absorption and Solubility in Water

For the water absorption (A) and solubility (S) tests, five disk-shaped samples (15 mm in diameter and 1 mm in height) were prepared for each material under investigation. The samples were light-cured for 20 s at five points using an LED light-curing lamp (Guilin Woodpecker Medical Instruments Co., Guilin, China). After light-curing, the samples were stored in a desiccator for 24 h to ensure complete drying.

Each sample was weighed multiple times until a constant weight was achieved, recorded as the initial weight (m_1_). The diameter and thickness of each sample were accurately measured at three distinct points using a digital caliper. The average radius (r) and thickness (h) were then utilized to calculate the volume (V) of each sample with the formula V = πr^2^ h [mm^3^].

Subsequently, the samples were immersed in 50 mL of distilled water in individual polyethylene containers and maintained in a thermostatically controlled water bath at 37 °C (±2 °C) for 7 days. After this period, the samples were carefully removed using tweezers, gently dried with filter paper, and exposed to air for 15 s. Each sample’s weight was measured three times, 1 min after removal from the water, with the average recorded as m_2_. The samples were then returned to the desiccator until reaching a constant weight, noted as m_3_.

The water absorption (A) and solubility (S) were calculated using the following equations:

A = (m_2_ − m_3_/V);

S = (m_1_ − m_3_/V).

Here,

m_1_ = initial weight of the sample before immersion in distilled water;

m_2_ = weight of the sample after immersion in distilled water;

m_3_ = weight of the dried sample after desiccation;

V = volume of the sample.

### 2.3. Fluoride Ions Release Test

Five specimens were prepared for the fluoride ion release test using the same procedure as for the water absorption and solubility tests. The samples were immersed in 45 mL of double-distilled water combined with 5 mL of TISAB III buffer (Total Ionic Strength Adjustment Buffer, concentrated solution, HI 4010-06, Hanna Instruments (Woonsocket, RI, USA) and placed in a thermostatically controlled water bath at 37 °C. Measurements were taken daily for the first 3 days, with a final measurement on day 7. After each measurement, the specimens were returned to their original polyethylene containers and stored in the thermostatically controlled bath at 37 °C.

Fluoride ion concentrations were measured with a fluoride ion-selective electrode (Combination Fluoride Electrode HI 4110, equipped with HI 7075 electrolyte for the reference electrode, Hanna Instruments). Before testing, the electrode was calibrated using a series of standard solutions containing fluoride concentrations ranging from 10^−6^ to 10^−2^ mol/L, prepared from a 1 M NaF stock solution (Merck). These standard solutions generated a calibration curve.

All solutions, including those used for calibration and sample analysis, were prepared in 50 mL of double-distilled water mixed with TISAB III buffer at a 45:5 ratio and maintained at 37 °C (±2 °C). Fluoride ion release was quantified and reported in ppm.

### 2.4. FTIR Analysis and Degree of Conversion

The infrared spectra of methacrylic monomer mixtures and their corresponding copolymers were obtained using a Jasco FTIR 610 spectrophotometer operating from 400 to 4000 cm^−1^ and equipped with an ATR (Attenuated Total Reflection) device. Spectra were recorded one hour after light-curing disk-shaped samples (4 mm × 0.5 mm). The curing process was performed using an LED E lamp with 20 s exposures on each side of the sample.

The absorbance baseline of each spectrum was recorded and then subtracted from the peak value at the corresponding wavenumber. The percentage of reacted double bonds, known as double bond conversion (C%), was calculated using the following formula:

C% = {1 − ([A_met_/A_arom_]_x_ copolymer/[A_met_/A_arom_]_x_ monomer)} × 100

where A_met_ is the absorption intensity of the C=C double bond in the monomer or polymer and A_arom_ is the absorption intensity of the C-C bond in the aromatic cycle.

The absorption band at 1635–1640 cm^−1^, corresponding to the stretching vibrations of the C=C double bond, was analyzed to determine the unreacted methacrylate groups.

The absorption band of the phenyl group at 1605–1610 cm^−1^ (for samples NB6 and NB6F) served as an internal standard. For samples NB3 and NB3F, the absorption band at 1531.2 cm^−1^, corresponding to the stretching vibrations of N-H bonds, was used as an internal reference. The ratios of the absorbance intensities for C=C/C-C, C=C/N-H, and C=C/C=O were compared before and after polymerization.

### 2.5. Residual Monomers

After completing the absorption and solubility tests, the storage media (10 mL of distilled water) in which the samples were immersed for 7 days were collected, frozen, and then lyophilized in a Lyophilizer Model Alpha 1-4 LDPLUS (Martin Christ Gefriertrocknungsanlagen GmbH, Osterode am Harz, Germany) until all the liquid was removed. The residue, containing the extracted residual monomers, was resuspended in 2 mL of acetonitrile, filtered through 0.45 μm PTFE filters, and subsequently analyzed using High-Performance Liquid Chromatography (HPLC).

HPLC analyses were performed using a Jasco HPLC chromatograph (Jasco, Tokyo, Japan), equipped with an HPLC pump (Model PU-980), a ternary gradient unit (Model LG-980-02), a column thermostat (Model CO-2060 Plus), a UV/VIS detector (Model UV-975), and an injection valve with a 20 µL sample loop (Rheodyne, CA, USA). A Hamilton Rheodyne syringe (50 mL) was used for manual injections. System control and data analysis were performed using ChromPass v 1.8 Software.

The analytes were separated on a Lichrosorb RP-C18 column (25 × 0.46 cm) at a column temperature of 21 °C. The mobile phase consisted of acetonitrile (A, HPLC grade) and ultrapure water (Milipore, Burlington, MA, USA), with the following gradient elution method: 0–15 min, linear gradient 50–80% acetonitrile; 15–25 min, linear gradient 80–50% acetonitrile. The flow rate was set at 0.9 mL·min^−1^, and the injection volume was always 20 µL. UV detection was performed at 204 nm to monitor the elution of analytes, including Bis-GMA, TEGDMA, HEMA, and UDMA, as they exhibit significant absorption at this wavelength. Stock solutions of reference compounds for Bis-GMA, TEGDMA, and UDMA (1 mg·mL^−1^) were prepared in acetonitrile and stored at 4 °C. Linearity of response was established with four concentration levels, yielding a regression factor (R^2^) of 0.998.

### 2.6. Cell Viability

The study was performed on an oral cell line of commercial human dysplastic oral keratinocytes (DOK, ECCAC 94122104, purchased from Sigma Aldrich, St. Louis, MI, USA). DOKs were cultured in DMEM (Dulbecco’s modified Eagle’s medium) supplemented with 0.5 µg/mL hydrocortisone (Sigma Aldrich, St. Louis, MI, USA), 5% FCS (fetal calf serum), streptomycin, penicillin, and amphotericin (all from Biochrom AG, Berlin, Germany), under standard conditions. The medium was changed twice a week.

For cell viability assessment, cells were cultivated in 96-well plates at a density of 104/well, incubated for 24 h, and then exposed to extracts of each experimental sample for 24 h. The extracts were prepared by incubating solid disk samples (thickness 1 mm) for 24 h at 37 °C, completely submerged in complete medium. Extraction was performed using 3 cm^2^/mL of each experimental material and of corresponding Icon^®^ samples, treated in the same way, in accordance with ISO 10993-12:2012 guidelines [17]. The extracts were used immediately for cell treatment without dilution. After exposure, cells were washed and viability was measured using the CellTiter 96^®^ AQueous Non-Radioactive Cell Proliferation Assay (Promega Corporation, Madison, WI, USA). The amount of formazan produced by viable cells was measured colorimetrically at 540 nm using the Spectra Max ID3 ELISA plate reader (Molecular Devices LLC, San Jose, CA, USA). Data (*n* = 3) were expressed as a percentage of untreated controls. Cells exposed to medium served as controls. The toxicity limit was set at 70% of the untreated control value.

### 2.7. Statistical Analysis

Statistical analysis was performed using GraphPad Prism 8 and Origin 2019b Graphing & Analysis software. Summary statistics were reported for all variables. Continuous variables were presented as mean and standard deviation. Comparisons between groups were conducted using the ANOVA test with the Tukey test for multiple comparisons. Mean differences and the 95% confidence intervals for multiple comparisons were reported. The significance level was set at α = 0.05.

## 3. Results

### 3.1. Flexural Strength

**Figure 1:**
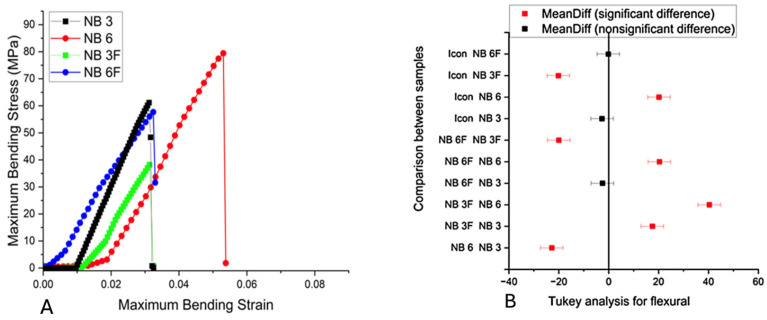
(**A**) Flexural strength: flexural strength of samples until fracture; (**B**) Tukey analysis of the flexural strength.

**Figure 2:**
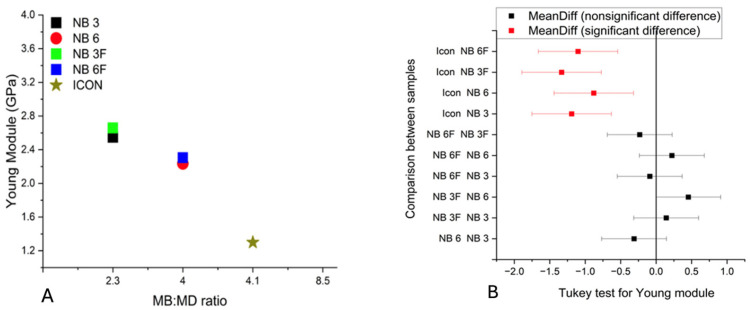
(**A**) The value of Young’s modulus of elasticity as a function of the ratio between the base monomer and the dilution monomer; (**B**) Tukey analysis of the modulus of elasticity.

The mechanical properties of the materials are summarized in Table 2. Figure 1A,B illustrate the flexural strength of the samples measured up to fracture and the results of the Tukey post hoc analysis, while Figure 2A,B present Young’s modulus of elasticity as a function of the base-to-diluent monomer ratio, including the results of the Tukey post hoc analysis. The lowest resistance was recorded for NB6, followed by the NB6F samples based on urethane and diluent monomers. The samples containing fillers exhibited higher flexural strength than those without fillers (NB3F > NB3; NB6F > NB6), with strength increasing by 20% in both cases. Significant statistical differences were found between the experimental groups (with and without fillers), as indicated in Figure 1B (*p*-value < 0.001).

For Young’s modulus of elasticity, the ranking was consistent across both groups: NB3F > NB3 and NB6F > NB6.

Data show that a higher proportion of the base monomer relative to the dilution monomer increased strength and modulus of elasticity. The UDMA monomer provided greater elasticity in the resin compared to the Bis-GMA resin samples. The addition of silanized nanoparticle fillers in the experimental resins enhanced the modulus of elasticity in both cases. Statistical analysis showed no significant differences in elasticity among the experimental groups (*p*-value = 0.07), particularly between those with and without fillers. However, when comparing the results of the four experimental samples to the commercial material sample, Icon^®^, differences can be observed, as illustrated in Figure 2A,B.

### 3.2. Absorption and Solubility in Water

**Figure 3:**
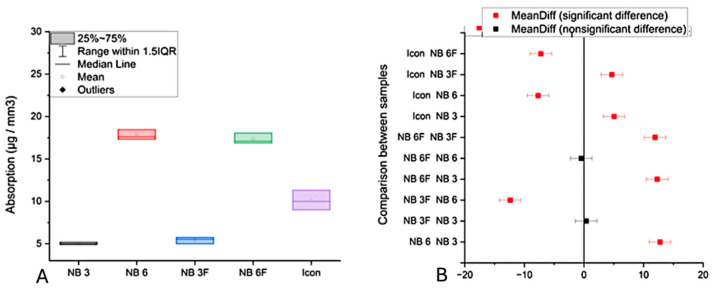
(**A**) The samples’ absorption values (µg/mm^3^); (**B**) Tukey analysis of absorption values.

**Figure 4:**
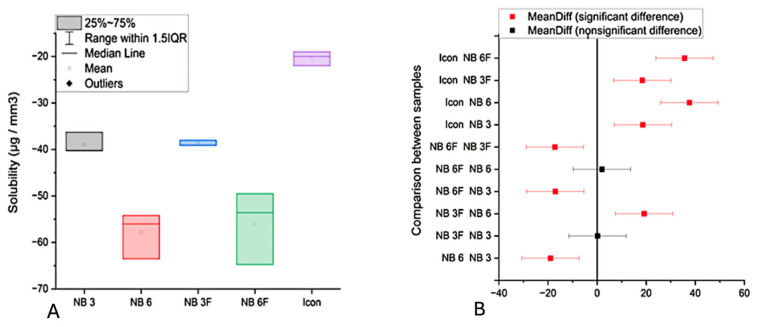
(**A**) The solubility value (µg/mm^3^) of the samples; (**B)** Tukey analysis of the solubility values.

Results for water solubility and absorption are presented in Figure 3A and Figure 3B, and Figure 4A and Figure 4B, respectively. Water absorption values ranged from 5.42 µg/mm^3^ for NB3 (TEGMA/UDMA) to higher levels for other materials. The lowest absorption (5.42 µg/mm^3^) was recorded for NB3, likely due to the hydrophobic nature of TEGMA. NB6 and NB6F, which contain Bis-GMA, showed significantly higher water absorption (16–18 µg/mm^3^) than NB3.

The results were analyzed over 7 days. The statistical analysis revealed that the absorption and solubility values differed significantly between the materials (*p* < 0.0001). The solubility values showed a similar trend, with significant differences observed among the groups (Figure 4B).

### 3.3. Amount of Released Fluoride Ions

**Figure 5:**
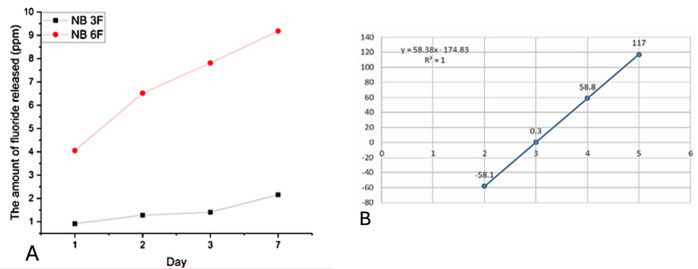
(**A**) The amount of fluoride released over time for each filled sample; (**B**) the calibration curve.

Figure 5A shows the fluoride ion release data and Figure 5B shows the calibration curve. The results were analyzed using a One-Way ANOVA test, followed by post hoc comparisons between the samples over the seven days. Significant differences were found among the samples (*p* = 0.00283) across all three days of fluoride release measurements.

NB3F (UDMA/TEGMA) released the least amount of fluoride, with a cumulative release of 2.15 ppm by day 7. In contrast, the NB6F sample containing Bis-GMA released a significantly higher cumulative amount of fluoride (9.18 ppm) by day 7.

### 3.4. Degree of Conversion

**Figure 6:**
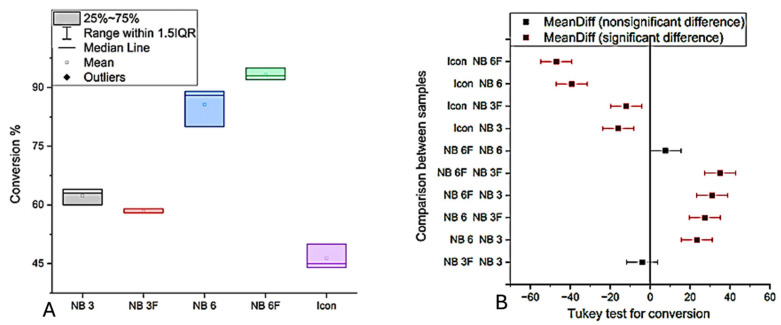
(**A**) Conversion of copolymers one hour after polymerization (%); (**B**) Tukey analysis of conversion.

FTIR spectroscopy was used to determine the degree of conversion (DC) of the experimental copolymers by comparing the absorbance of reactive functional groups before and after polymerization. The measured DC values varied depending on resin composition and filler incorporation (Figure 6A). All formulations exhibited relatively high conversion degrees, consistent with values reported in the literature for Bis-GMA/TEGDMA-based systems, where a 10/90 wt% ratio typically yields a DC of approximately 76%.

NB6F samples, which included fluorohydroxyapatite and BaF_2_ glass filler, exhibited a slightly higher degree of conversion compared to NB6 samples without filler (Figure 3). Specifically, the Bis-GMA-containing samples (NB6 and NB6F) achieved a conversion degree of 80% after one hour of polymerization, with a slight increase in conversion observed when the filler was incorporated into the organic matrix (NB6F). Statistically significant differences (*p* < 0.0001) were observed between the groups; however, no significant differences were detected between the NB3 and NB3F groups or between the NB6 and NB6F groups (Figure 6B).

These results indicate that incorporating fluoride into the filler within the polymeric matrix only slightly enhanced the degree of conversion, with a more pronounced effect observed in the NB6F group.

### 3.5. Measurement of Residual Monomers

**Figure 7:**
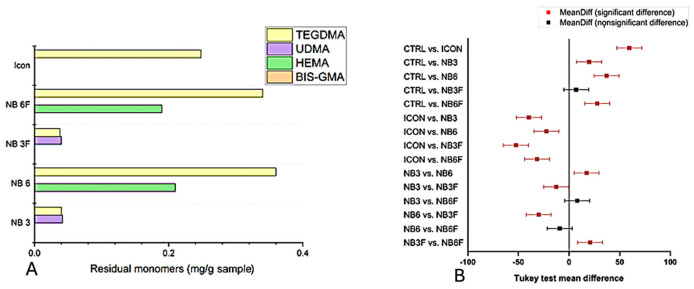
(**A**) Quantitative values of the residual monomer; (**B**) Tukey analysis of the quantity of residual monomers.

Aliphatic TEGDMA monomer was found in all examined samples (Figure 7A). The NB6 sample had the highest TEGDMA concentration at 360.92 ppm. Similarly, the NB3 sample exhibited the highest extraction of UDMA at 42.244 ppm. NB6 also showed the highest HEMA concentration at 210.294 ppm. In contrast, Bis-GMA did not display any characteristic peaks in the HPLC analysis.

The total quantities of residual monomers extracted from the samples were ranked in descending order: NB6 > NB6F > Icon^®^ > NB3 > NB3F. The lowest residual monomer content (75.4 ppm) was found in the NB3F sample, while the highest content (560 ppm) was recorded for NB6. Pairwise comparisons conducted using the Tukey test (Figure 7B) showed significant differences among groups (*p* < 0.0001), confirming the variability of monomer extraction.

### 3.6. Biocompatibility Assay

**Figure 8:**
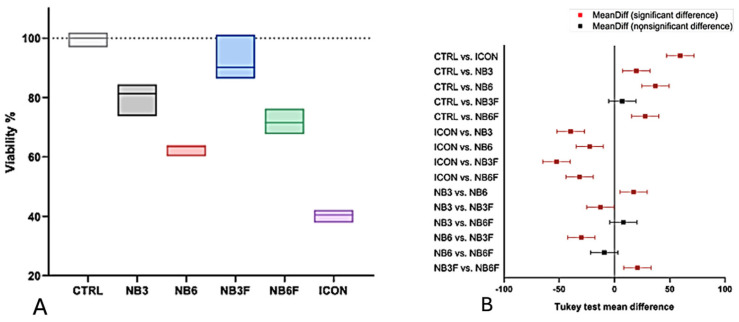
(**A**) Viability of the cells treated with experimental and commercial materials. Data are expressed as a percentage of the untreated control; results are presented as the mean (*n* = 3) ± STDEV. (**B**) Tukey test for pairwise comparisons.

The biocompatibility of the materials was investigated by assessing their impact on the viability of a human oral cell line in vitro (Figure 8A). Icon significantly decreased cell viability to 40.5% as compared to untreated controls (*p* < 0.001, mean difference 59.4). The experimental materials were better tolerated by the cells when compared to Icon. Specifically, NB6 and NB6F exhibited mild toxic effects on DOKs, with viability rates of 63.7% and 71.5%, respectively, which were significant compared to the untreated control (*p* < 0.001), but less severe compared to Icon. NB3 and NB3F exhibited the highest cell viability (81.3% and 90.2%), with no significant difference compared to the control (*p* = 0.441) in the case of NB3F (Figure 8B).

## 4. Discussion

Infiltrating white spot lesions with low-viscosity dimethacrylate resins is a beneficial treatment option for early-stage caries [18]. Unlike the traditional method of sealing occlusal pits and fissures, infiltrating agents are designed to penetrate the entire porous structure of the lesion without filling the free surface volume. This characteristic is vital to overcome the space challenges encountered in marginal applications. In this study, Icon^®^ was used as a reference control because it is a commercially available infiltration material, ensuring the consistency and relevance of the results.

This study’s novelty lies in the formulation and characterization of infiltration resins using a mixture of hydroxyapatite and glasses. In our research, the samples containing UDMA showed increased flexural strength, with significant differences when compared to samples containing the basic monomer Bis-GMA, which had a higher proportion of the diluent monomer. UDMA has been highlighted in the literature as a more flexible and reactive monomer compared to Bis-GMA, exhibiting high flexural strength (up to 133.8 MPa) and hardness among homopolymers. Furthermore, combinations of UDMA with TEGDMA are reported to form more homogeneous polymer networks, achieving degrees of conversion exceeding 70%. This aligns with previous studies indicating that TEGDMA/UDMA formulations improve mechanical properties relative to those with higher diluent content [19,20]. At the same time, the literature data highlight the dominant role of Bis-GMA in determining composite stiffness, with Young’s modulus values consistently higher than those of UDMA or TEGDMA. Such differences suggest that the selection and ratio of monomers can strongly influence the balance between rigidity and toughness in dental composites [21], and that careful optimization of monomer combinations, including UDMA and low-viscosity comonomers, is key to achieving both high conversion and favorable mechanical performance. In addition, incorporating nanofiller into the infiltrates’ composition can act as both a dispersion phase and a reinforcing agent, enhancing their mechanical and aesthetic properties. Nanoparticles with high crystallinity also promote greater light transmission, improving polymerization efficiency. These components directly contribute to the materials’ flexural strength and elastic modulus, improving their mechanical performance [22].

Absorption and solubility are fundamental characteristics of materials that can impact their strength and durability. A low surface free energy, typical of highly hydrophobic materials, can reduce bacterial adhesion. For infiltrates primarily composed of TEGDMA, water absorption increases because of its polar ether bonds. Hence, adding monomers such as Bis-GMA or UDMA should be considered to maintain hydrolytic balance, depending on the specific application. As other studies have also shown, adding the hydrophobic monomer UDMA to the TEGDMA mixture enhances mechanical properties while maintaining penetrability [19]. Our findings are consistent with previous studies, which show that the main component of Icon^®^, TEGDMA, is prone to water sorption, hydrolysis, and enzymatic degradation, which may negatively impact its long-term stability and contribute to discoloration and increased biofilm accumulation [22].

Hydrolytic degradation of resin-based materials reduces their physical–mechanical properties, thereby affecting their longevity. The inclusion of nanoparticles may enhance the materials’ absorption, potentially due to the increased hydrophilicity they provide, depending on their specific properties [23]. The quality of the polymeric matrix primarily affects the durability of infiltrates, and incorporating an inorganic nanofiller into its composition can decrease polymerization shrinkage and hydrolytic degradation [9].

Previous research indicated a decrease in the degree of conversion when TEGDMA content exceeds 70 mol% [8]. Icon^®^ contains over 90% wt of TEGDMA, which explains its lower degree of conversion. A low degree of conversion may be linked to a higher percentage of residual monomers, which could lead to hydrolytic degradation and, ultimately, a decrease in the material’s mechanical properties [24].

Our data show that the properties of the synthesized materials are significantly influenced by their chemical composition. Although both NB3F and NB6F materials contain the same percentage of HAF-BaF_2_ powder, the material without UDMA (NB6F) released more fluoride than the one with UDMA (NB3F). This phenomenon could be attributed to UDMA’s moderate water sorption [18] and to ion diffusion, which depends on factors such as the polymer matrix’s permeability and the role of water in facilitating ion transport within the storage medium [7]. The fluoride release behavior observed for the NB3F and NB6F groups may have important implications for their remineralization potential. Fluoride ions are known to enhance enamel remineralization by promoting fluorapatite formation and inhibiting demineralization. The relatively low fluoride release from NB3F suggests a limited contribution to remineralization when compared to the other experimental groups. In contrast, the significantly higher cumulative fluoride release exhibited by NB6F indicates a greater potential to support remineralization processes over time. This enhanced release may be attributed to differences in resin matrix composition and filler content, particularly the presence of Bis-GMA, which can influence water uptake and ion diffusion. Compared with other groups, NB6F may therefore offer improved remineralizing capability due to its sustained fluoride release, while still maintaining the functional characteristics of a resin infiltrant.

The materials containing UDMA, NB3, and NB3F exhibited the lowest water sorption, which may be explained by the long hydrocarbon chains and urethane groups present in UDMA, contributing to its water resistance [25].

Despite the minimally invasive nature of the infiltration technique, the presence of HEMA (hydrophilic) and TEGDMA (lipophilic) can lead to pulp inflammation by diffusing through dentin tubules (HEMA) and penetrating the lipid layer (TEGDMA) of the cells of the dental pulp. Therefore, reducing the polymerization time may increase cellular apoptosis due to the release of residual monomers, which can penetrate surrounding tissues and exert cytotoxic effects [26].

The lower residual monomer levels in NB3 and NB3F may also explain why these two materials exhibited the highest viability. Residual monomers can induce toxic effects in various cells, potentially triggering apoptosis and genotoxic events, inhibiting cell function, and impacting the innate immune system, all due to their high chemical reactivity [27].

Compared with our findings demonstrating the cytotoxic effects of the TEGDMA-based infiltrant, previous studies have suggested that such infiltrates can induce cytotoxicity and inflammatory reactions, depending on the degree of polymerization. Our data support this hypothesis, showing a significant decrease in cell viability, which may be attributed to residual monomers and their high chemical reactivity [27]. In particular, TEGDMA is readily released from the resin matrix, contributing to cytotoxic effects, and is susceptible to hydrolysis in the oral environment, which may further affect cellular responses [28]. TEGDMA is a low-molecular-weight monomer characterized by high mobility and surfactant properties, which allow it to pass through cell membranes more easily than bulkier monomers such as Bis-GMA. Once inside the cell, TEGDMA is susceptible to hydrolysis and enzymatic degradation in the oral environment, further exacerbating the cellular response [29,30]. Among the materials included in our study, Icon^®^ exhibited the most substantial decrease in cell viability, which can be attributed to its primary component, TEGDMA, and is consistent with findings from other studies. TEGDMA is one of the most cytotoxic dental resin monomers due to its high hydrophilicity and release into the oral cavity, with its cytotoxic effects consistently demonstrated across various cell cultures [22]. After entering the cell, TEGDMA undergoes hydrolysis and enzymatic degradation in the oral environment, which can amplify the cellular response [31]. According to ISO 10993-5:2009 [32], a cell viability below 70% of the control indicates cytotoxicity. In our study, Icon^®^ demonstrated a significant reduction in cell viability, with values reaching 40.5%, further emphasizing its cytotoxic effects.

Resin infiltrants are primarily designed to improve the visual appearance of white spot lesions (WSLs) by masking their opacity. However, the current evidence is limited regarding several critical factors that influence their clinical effectiveness. Specifically, data on the penetration depth of the resin into the demineralized enamel are sparse, yet this parameter directly affects the stability and durability of the aesthetic improvement [33]. Similarly, the degree of refractive index matching between the resin and the surrounding enamel has not been thoroughly quantified, despite its importance in achieving optimal optical blending. Without comprehensive evaluation of these aspects, including long-term aesthetic performance, the true clinical benefit of resin infiltration remains incompletely understood. Future studies should therefore address these dimensions to provide a more holistic assessment of resin infiltrants in the management of WSLs [34].

The present study evaluated the experimental materials over short periods (up to 7 days) under static in vitro conditions, which do not fully replicate the dynamic oral environment. Factors such as temperature fluctuations, acidic challenges, enzymatic activity, and mechanical wear can also significantly influence the long-term performance and durability of resin infiltrants. The current findings primarily reflect initial physicochemical properties and biocompatibility. Future studies will include more comprehensive simulations of oral conditions, including thermocycling, pH cycling, and mechanical loading, to further assess long-term functionality and clinical relevance.

Our study evaluated in vitro cytotoxicity using DOK cell lines and observed differences among samples. However, due to the limitations of in vitro models, these results cannot be directly extrapolated to in vivo conditions, where factors such as the tissue microenvironment and immune response may significantly influence the cytotoxicity of these materials.

## 5. Conclusions

Within the limitations of the study, our findings indicate that modifying the chemical structure of the infiltration materials affects their physicochemical and mechanical properties.

The addition of inorganic nanofillers (NB3F and NB6F) increased the degree of conversion of the materials compared to those without the nanofillers (NB3 and NB6). Furthermore, the nature of the polymeric matrix also influenced the degree of conversion, the level of residual monomer release, and, consequently, the hydrolytic balance. Although a lower percentage of Bis-GMA was used as the base monomer in the polymeric matrix of the NB6 and NB6F materials, one hour after polymerization, the conversion was slightly higher than that of the NB3 and NB3F groups, which used UDMA as the base monomer in their polymeric matrix.

Because of their greater cell viability, the newly synthesized experimental resins may provide better biocompatibility than Icon^®^.

All these variations must be considered in relation to the intended clinical application to achieve optimal results.

## Figures and Tables

**Table 1:** Composition of nanobiomaterials.

Samples	Organic Phase (%)				Inorganic Phase (%)
	TEGDMA	UDMA	HEMA	Bis-GMA	HAF-BaF_2_ Glass
NB3	70	30	-	-	-
NB6	65	-	15	20	-
NB3F	67	28	-	-	5 (2 + 3)
NB6F	62	-	14	19	5 (3 + 2)
Icon^®^	Metacrylate-based resin TEGDMA (70–95%), initiators, additives < 2.5%				

TEGDMA = triethylene glycol dimethacrylate; UDMA = urethane dimethacrylate; Bis-GMA = 2,2-bis [4-(2-hydroxy-3-methacryloxypropoxy) phenyl] propane (synthetized at ICCRR-UBB); HEMA = hydroxyethyl methacrylate. Glass and HAF= fluor-hydroxyapatite (synthetized at ICCRR-UBB).

**Table 2:** The mean values of the maximum force recorded until fracture ± standard deviation (N), modulus of elasticity ± standard deviation (GPa), stress at fracture ± standard deviation (MPa), and rigidity ± standard deviation (N/mm).

Group	Maximum Load (N)	Young Modulus (GPa)	Force (MPa)	Rigidity (N/mm)
NB3	16.155 ± 1.840	2.549 ± 0.937	62.114± 1.8991	22833 ± 4132
NB6	12.376 ± 1.068	2.235 ± 0.296	38.848± 3.5482	24792 ± 1790
NB3F	23.454 ± 0.704	2.658 ± 0.549	79.951± 6.5621	24458 ± 1093
NB6F	18.071 ± 3.804	2.305 ± 0.339	58.114 ± 9.4110	20817 ± 1803

## Data Availability

The original contributions presented in this study are included in the article. Further inquiries can be directed to the corresponding author.
